# Resistance exercise snacks improve muscle mass in female university employees: a prospective, controlled, intervention pilot-study

**DOI:** 10.3389/fpubh.2024.1347825

**Published:** 2024-02-06

**Authors:** Tom Brandt, Christian Thomas Lothar Schwandner, Annette Schmidt

**Affiliations:** Institute of Sports Science, University of the Bundeswehr Munich, Neubiberg, Germany

**Keywords:** workplace health, muscle mass, strength, functional capacity, balance, resistance training, aging

## Abstract

**Background:**

Although resistance training (RT) is essential to preserve musculoskeletal fitness and maintain a healthy, independent life into old age, few women perform RT. We investigated whether resistance exercise snacking (RES) could be an efficient training approach for the workplace health promotion (WHP) to minimize barriers for participation and facilitate RT in women in order to improve musculoskeletal fitness.

**Methods:**

This pilot-study followed a prospective, controlled intervention design. Female employees with sedentary occupations doing RT on less than 2 days/week before study participation were included. Participants self-selected for either intervention (IG) or control group (CG). While the IG [*N* = 15, mean age 42.1 (*SD* = 11.1) years] did 10 min of RES on working days for 12 weeks, the CG [*N* = 15, mean age 49.9 (*SD* = 9.7) years] was instructed to maintain their habitual physical activity. Primary endpoint was change in muscle mass. Secondary endpoint was change in maximum isometric strength. Balance, cardiovascular fitness, perceived health, and general life satisfaction was assessed for exploratory purpose. Measurements were taken before and after the intervention.

**Results:**

12 participants of IG and 14 of CG completed the study. Muscle mass improved significantly more in the IG [+0.42 (SD = 0.54) kg] compared to the CG [−0.16 (*SD* = 0.51) kg] (*p* = 0.01, *ƞ^2^_p_* = 0.24). Strength did not change significantly between groups. Nevertheless, there was a trend for greater improvements in the IG compared to the CG for trunk extension, trunk flexion, and upper body push but not upper body pull. Regarding exploratory endpoints, no significant between-group changes were found. Despite their poor fitness, both groups perceived their health as good and had high life satisfaction before and after the intervention.

**Conclusion:**

RES could be an effective approach for the WHP to promote RT in inactive women with sedentary occupations and improve their muscle mass.

## 1 Introduction

Aging is accompanied by a gradual decline in measures of musculoskeletal fitness (e.g., spinal motor neurons, bone density, strength, power, muscle fiber number, and muscle fiber size) (1, 2). Lexell et al. (3) have shown that age-associated loss of muscle mass could already begin at the age of 25 and accelerates thereafter resulting in a 10% loss of muscle area at the age of 50. According to Doherty, this is particularly relevant since age-related loss of muscle mass is the most contributing factor to the decline of muscular strength (4). This could impair the ability to be physically active and perform everyday tasks. In the long-term, a downward spiral of physical inactivity and progressive loss of functional capacity may emerge which could have detrimental effects on other physiologically important systems (e.g., cardiovascular system) and overall health status (5, 6). The most effective non-pharmacological method to increase muscle mass, strength, and power across the lifespan is resistance training (RT) (7). Accordingly, the World Health Organization (WHO) and the American College of Sports Medicine (ACSM) recommend to perform RT including all major muscle groups at least twice a week (8, 9). This recommendation is followed by only 10–30% of adults, with women engaging in even less RT than men (10–12). However, RT could be especially beneficial for women as they have less skeletal muscle mass, experience an earlier strength loss, and a greater decline in muscle quality compared to men (4, 13). Common barriers for RT participation include lack of time, limited access to equipment and facilities as well as low competence or skill (7, 14, 15).

A novel approach that may limit these barriers and facilitate RT participation in women are “resistance exercise snacks” (RES). RES are brief (e.g., ≤ 15 min) RT bouts executed frequently throughout the week or day (e.g., 5–7 RES per week) at low intensities (e.g., bodyweight) with minimal to no equipment (e.g., resistance bands or portable weights) (7). In this regard, RES could provide a convenient, health-promoting strategy especially for women with low physical fitness that do not perform any other form of training. RES could help these women to integrate RT into everyday life in order to fulfill physical activity recommendations, for example in between periods of prolonged sitting. This notion was supported by previous studies in individuals with type 2 diabetes. These studies indicated that RES could be an effective method to decrease resting blood pressure, plasma noradrenaline, acute postprandial glucose, insulin, C-peptide, and triglyceride responses when performed to interrupt prolonged sitting (16, 17). This is of particular interest in the area of workplace health promotion (WHP), considering the high prevalence of predominantly sedentary occupations among women in modern societies (18). Consequently, incorporating RES into the WHP could simultaneously reduce the detrimental health effects of prolonged sitting during working hours and increase RT participation while leveraging the efficient structures to reach large groups and social network of the workplace (19–21).

However, research examining explicitly the efficacy of RES to improve muscle mass, strength, power, or functional capacity is still sparse and partly contradictory. In a pilot study, Perkin et al. investigated whether a 4-week RES intervention improves measures of muscle function in healthy older adults (*N* = 20). The intervention group (IG) executed 5 min of RES twice per day, whereas participants of the control group (CG) had to maintain their habitual physical activity. Although the IG showed greater improvements than the CG in leg pressing muscle force (IG: +5%, CG: − 2%) and power (IG: + 6%, CG: −2%), there was no significant difference in change between groups. Additionally, a positive change was observed for lean leg mass (+1%) and thigh muscle cross-sectional area (+2%) in the IG, with no significant changes between groups neither. Significant changes between groups occurred only in a 60-s sit-to-stand test in favor of the IG (22). Contrarily, Fyfe et al. compared different RES protocols [4 weeks intervention; groups: once (*N* = 9), twice (*N* = 10), and thrice (*N* = 9) RES per day, and habitual-activity control (*N* = 10)] in older adults but found no significant difference in change between groups in a 30-s sit-to-stand test. Nevertheless, participants rated the RES intervention as enjoyable (75% reported a score ≥ 4 on a five-point Likert scale) and 82% responded that they would continue a similar training regimen after study completion (23). However, as the aforementioned studies were conducted in older adults, the intervention periods were short, the sample sizes small, and the analyses did not differentiate between women and men, it remains difficult to determine the longer-term effectiveness of RES in women in a workplace setting (22–24). Furthermore, none of the studies was done in a workplace setting.

Therefore, the aim of the current study was to examine the effects of a 12-week workplace health RES intervention on muscle mass and muscular strength in women with a predominantly sedentary occupation that did insufficient RT (< 2 RT sessions per week) prior study participation. For exploratory purpose, balance, cardiovascular fitness, perceived health, and general life satisfaction was assessed.

## 2 Materials and methods

### 2.1 Trial oversight

This pilot-study followed a prospective intervention design with control (CG) and intervention group (IG). Data were collected from March 2023 to August 2023. Participants self-selected for either IG or CG. The testing at baseline (t0) and after 12 weeks (t1) was done in the same manner for both groups. The CG was instructed to maintain their current activity level. The IG participated in a RES program for 10 min each working day. Integration of the study into the WHP of the University of the Bundeswehr Munich (UniBw M) enabled participants to take part during their working hours.

The Institutional Ethics Committee of the UniBw M approved the study protocol, ensuring that it conformed to the ethical guidelines of the 1975 Declaration of Helsinki. Informed consent was obtained from all subjects involved in the study (06/06/2023; EK UniBw M 23-43).

### 2.2 Participants

Female office workers of the central administration staff at the UniBw M (age = 18–65 years) participated in the study. Inclusion criteria were a mainly sitting or standing job and performing fewer than 2 RT per week. Women were excluded due to pregnancy or health issues that would preclude participation in regular exercise or the applied tests (e.g., severe injuries to the musculoskeletal system, osteoporosis, intervertebral disc damage, joint replacements, hypertension, and fresh scars). These criteria were checked via questionnaire at t0 and t1. Table 1 displays demographics and anthropometrics of initially assessed participants.

**Table 1:** Demographics and anthropometrics of initially assessed participants.

Variable	Control group	Intervention group	*p*
*N*	15	15	
Age (years)	49.9 (9.7)	42.1 (11.1)	0.05
Body mass (kg)	81.9 (14)	72.4 (14)	0.08
BMI (kg/m^2^)	28.9 (3.8)	26.9 (4.3)	0.19
Body fat (%)	40.6 (5.7)	35.4 (7.1)	**0.04**
Muscle mass (kg)	22.2 (3.7)	20.9 (3.3)	0.35

Age, body mass, BMI, body fat, and muscle mass of control and intervention group are given as mean (SD).

BMI, Body mass index; kg, kilograms; m, meters; and SD, Standard deviation.

The bold values indicate that the difference was statistically significant.

**Figure 1:**
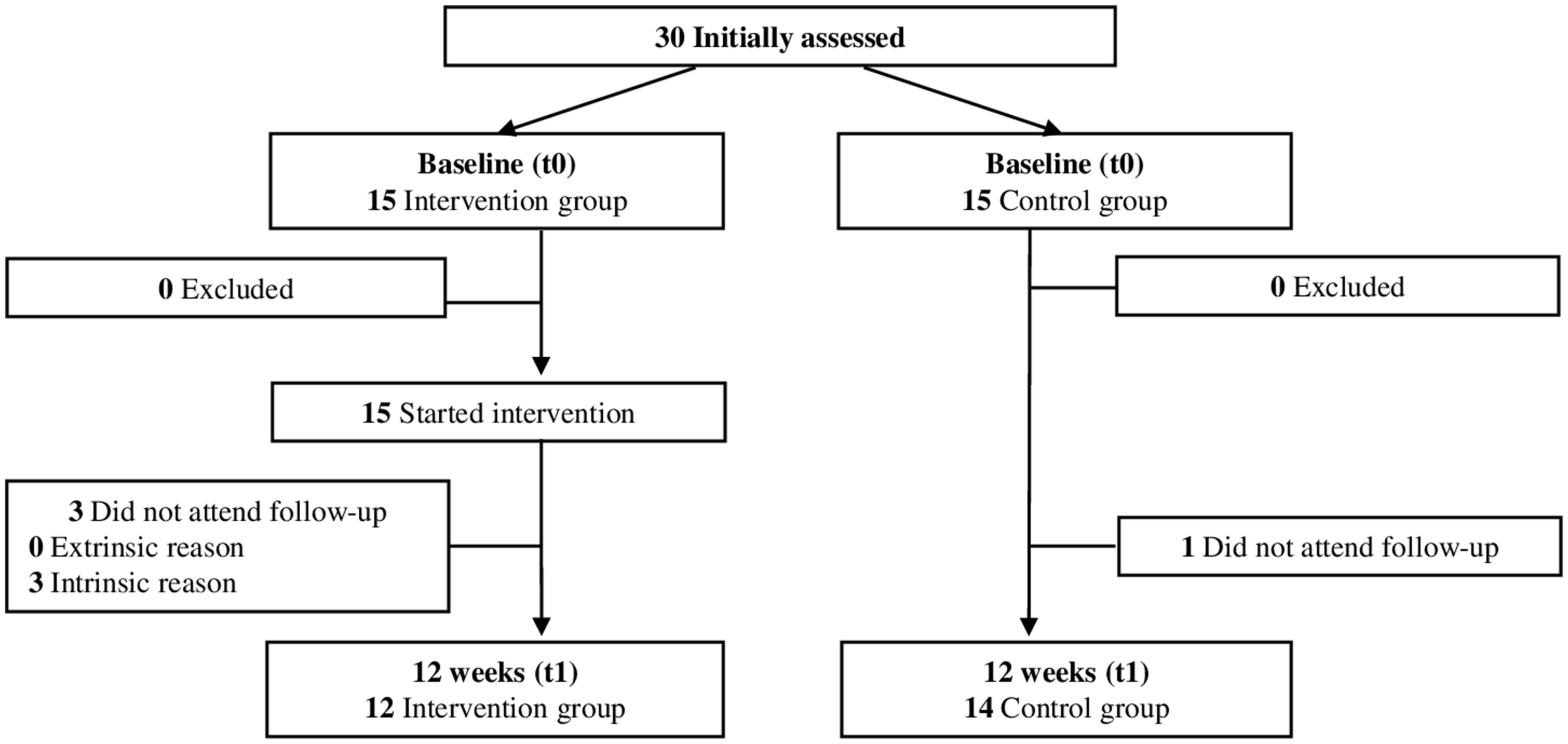
Participant flow over the course of the study.

The study was enrolled with 30 participants (IG: *N* = 15; CG: *N* = 15). After 12 weeks, three participants of the IG (20%; *N*_drop-out_/*N*_baseline_) and one of the CG (7%) dropped out of the study. All of them mentioned intrinsic reasons. Data sets are partly incomplete. One participant of the CG suffered from a minor wrist injury preventing her from performing the strength tests with the BC at t0. Another participant of the CG mentioned pain during the UPull at t0. Again, in the CG, one participant was unable to execute the YBT and 6MWT due to hip pain. Two participants of the IG did not perform the 6MWT according to the prescribed testing protocol and were therefore excluded from the analysis. No adverse events occurred during the intervention. Although participants were instructed to train on 5 days per week, one participant reported that she did 6 additional RES during the 12-week trial and one participant trained every day. Over the course of 12 weeks, a mean training attendance of 49.8 (17.1) sessions was documented. All participants that finished the study intended to continue the RES after completion. The participant flow is displayed in Figure 1.

### 2.3 Training intervention

For 12 weeks, the IG performed a 10-min RES on 5 days per week. The RES were designed to be carried out in proximity to the office and required minimal space and equipment. Each session consisted of strength- and mobility-enhancing exercises that were done for 30–60 s consecutively (e.g., squats, push-ups, lunges, good mornings, wall sits, side bends, standing knee raises, calve raises, standing leg lifts, cossack squats, and reverse flys). Participants were instructed to complete as many repetitions with correct form as possible in the given time. There was no rest between exercises. Participants performed the RES routine for 2 weeks before the exercise selection was changed. The first 2 days after a new RES routine was introduced, the participants performed the RES in small groups under supervision of a coach. When participants were unable to attend the group sessions (e.g., due to home office or business travel), the training plan and instructions were provided remotely by the coach. All participants followed the same program, but the exercises were scaled by the trainer if the participants were unable to perform the prescribed technique.

### 2.4 Endpoints and protocol

Primary endpoint of this study was the change in muscle mass from t0 to t1. Secondary endpoints were the changes in strength (maximum isometric strength in kg; Dr. WOLFF BackCheck® 617) (25, 26). Balance (Y-Balance test), body fat percentage, cardiovascular fitness (6-min walking test), perceived health, and general life satisfaction was assessed for exploratory purpose (27, 28).

Testing personnel and participants were not blinded. In the 24 h before the test sessions, the participants had to avoid any intense physical activity. The test sessions were done during working hours. The participants were instructed to maintain the same fluid and food intake on the test days and to attend both test sessions at the same time of day. The test sessions started with a questionnaire to assess medical history, physical activity, perceived health, and general life satisfaction of the participants. Thereafter, body composition, strength, balance, and cardiovascular fitness were measured.

#### 2.4.1 Height and body composition

Height and body composition were assessed in underwear. Height was measured with a SECA® 213 (seca GmbH & Co. KG, Hamburg, Germany) and body composition with a SECA® mBCA 515 scale (seca GmbH & Co. KG, Hamburg, Germany). Bosy-Westphal et al. validated the SECA® mBCA 515 against whole-body magnetic resonance imaging and found that the muscle mass determined by the SECA® mBCA was 97% (*R^2^* = 0.97) consistent with that of the whole-body magnetic resonance imaging (29).

#### 2.4.2 Strength

The Dr. WOLFF BackCheck® 617 (Dr. WOLFF® Sports & Prevention GmbH, Arnsberg, Germany) (BC) was used to assess maximum isometric strength in kilograms (kg) as it provides high enough test-/retest reliability and criteria validity to be used in scientific research (26). After participants were instructed, they attempted each movement three times. The best result was selected. Movements were executed in the following sequence: trunk extension (TE), trunk flexion (TF), upper body push (UPush), and upper body pull (UPull). All movements were performed with the participants standing upright in the BC. The participants were positioned in the BC using adjustable pads. The positions of the pads were noted in order to be set identically at t1.

#### 2.4.3 Balance

Balance was assessed with the Y-Balance test (Functional Movement Systems Inc., Chatham, VA, United States) (YBT). The YBT kit consists of a *y*-shaped PVC-pipe structure, a fixed central footplate, and three movable reach indicators that are shoved upon the arms of the *y*-shaped PVC-pipes. Each arm represents one reach direction (anterior, posteromedial, and posterolateral). The test required the participants to stand on the central platform with his hands placed on their hips and push the reach indicator along the given reach direction as far as possible without stepping from the platforms. Before the test, participants did 6 familiarization attempts for every reach direction. Afterward, participants were given 3 attempts per leg and reach direction of which the farthest was selected. The distance is given in centimeters (m) (30). Additionally, the leg length of the participants was measured to calculate the composite score for the right and left leg (31). The equation for the composite scores reads:


$$\text{Composite score}=\frac{\begin{array}{c} \text{anterior}\;\left(cm\right)+\text{posteromedial}\;\left(cm\right)\\ +\text{posterolateral}\;\left(cm\right) \end{array}}{\text{leg}\;\text{length}\;\left(cm\right)*3}*100$$

Composite scores are given in percent. The YBT has excellent inter and intra-rater reliability when applied to healthy adults (28).

#### 2.4.4 Cardiovascular fitness

The 6-min walking test (6MWT) is a method to monitor the cardiovascular and pulmonary performance below the anaerobic threshold. For this test, participants were instructed to walk a maximal distance within 6 min according to the standardized protocol (32, 33). Measuring the total distance covered by the participants is an efficient and inexpensive procedure to assess physical function and capacity to perform everyday activities (34). The distance is given in meters (m).

#### 2.4.5 Perceived health

The perceived health is an indicator for the objective health status and used in national as well as international health surveys (35). In the current study, we included a question of the minimum European health module, which is also recommended by the WHO (35, 36). Participants answered the German version of the question “How is your health in general?” and were given the response options “very good, good, fair, bad, and very bad.”

#### 2.4.6 General life satisfaction

The German version of the general life satisfaction short scale (L-1) is a validated, reliable measurement for general life satisfaction (37). Participants were asked to answer the question “The next question is about your general satisfaction with life. All things considered, how satisfied are you with your life these days?” on an 11-point scale ranging from “not at all satisfied” (0) to “completely satisfied” (10).

### 2.5 Statistical approach

For this study, exclusively women that did less than 2 RT sessions per week before study participation were included. Therefore, low baseline muscle mass and strength values, but high potential for improvements in these measures were expected (1). Due to the short intervention period and minimal dose of RT a medium effect for the primary endpoint was expected. Therefore, 12 participants per group were determined to achieve a power of at least 95% on a two-sided, 5% significance level. Based on previous research with similar designs, a dropout of 20% (*N*_dropout_/*N*_baseline_) was determined (22, 38). The effectiveness of the intervention regarding primary and secondary endpoints was determined by the difference in change between groups and analyzed via a mixed model ANOVA approach. Normal distribution was examined with Q-Q-plots and Kolmogorov–Smirnov test. Statistical significance was set at *p* ≤ 0.05. The same statistical approach was conducted for exploratory endpoints with interval scaled data. For ordinal scaled data (perceived health and general life satisfaction), the Wilcoxon test was performed to analyze the difference in change within groups.

Values for t0 and t1 as well as changes from t0 to t1 within groups are expressed as mean [standard deviation (SD)] in case of interval scaled data. Differences in change between groups are presented as mean (SD). Values for ordinal scaled data are expressed as median [inter quartile range (IQR)]. Effect sizes of primary, secondary, and exploratory endpoints with interval scaled data are given in partial ƞ^2^. For exploratory endpoints with ordinal scaled data Pearson’s r was calculated to give an estimate of the effect sizes. Data analysis was done with SPSS 29® (IBM SPSS, Armonk, NY, United States).

## 3 Results

### 3.1 Primary endpoint

**Figure 2:**
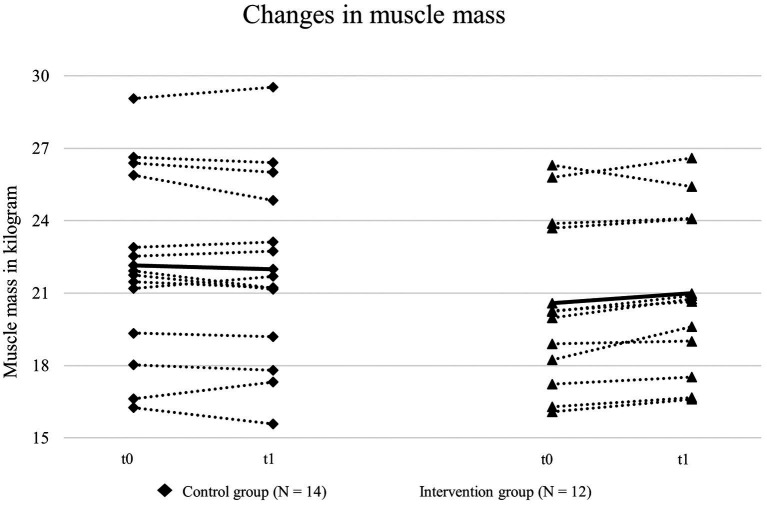
Muscle mass of intervention and control group before (t0) and after (t1) the intervention.

Mean muscle mass did not differ significantly between CG [22.2 (3.9) kg] and IG [20.6 (3.6) kg] at t0 (*p* = 0.31). Mean change in muscle mass from t0 to t1 was −0.16 (0.51) kg for CG and 0.42 (0.54) kg for IG. The difference in change between groups was significant 0.57 [0.15 - 1] kg, *p* = 0.01) at an effect size of *ƞ^2^_p_* = 0.24. The change in muscle mass from t0 to t1 is displayed in Figure 2.

### 3.2 Secondary endpoints

The CG had significantly higher maximum isometric strength values than the IG in the TE (*p* = 0.046) at t0. No significant differences were found for TF (*p* = 0.06), UPush (*p* = 0.71), and UPull (*p* = 0.28) at baseline. After 12 weeks, there was no significant difference in change between the IG and CG for TE, TF, UPush, and UPull. However, both groups showed higher strength values after 12 weeks for TE [IG: +10.9 (9.6) kg, CG: +5.1 (9.6) kg], TF [IG: +4.7 (8.8) kg, CG: +1.6 (6.3) kg], and UPush [IG: +10.2 (12.6) kg, CG: +6.6 (12.6) kg]. In the UPull, the CG reached higher strength values after the intervention period while the IG decreased [IG: −0.6 (12.1) kg, CG: +6.3 (8.7) kg]. The greatest change in the IG occurred in the TE (+33%) followed by UPush (+18%), TF (+16%), and UPull (−1%). Contrary, the CG improved most in the UPull (+14%) followed by UPush (+12%), TE (+11%), and TF (+4%).

### 3.3 Exploratory endpoints

At baseline, mean YBT scores of the IG were significantly higher for YBTl but not YBTr when compared to the CG (YBTr: *p* = 0.06, YBTl: *p* = 0.047). After 12 weeks, the IG showed greater improvements than the CG for YBTr [IG: 4.1 (4.3) %, CG: 3.4 (6.6) %] and YBTl [IG: 3.5 (9.3) %, CG: 3.4 (6.2) %] scores. Between-group changes in YBTr (*ƞ^2^_p_* = 0.002, *p* = 0.84) and YBTl scores (*ƞ^2^_p_* = 0, *p* = 0.99) were not significantly different.

Before the intervention, body fat percentage differed significantly between IG [35.3 (6.6) %] and CG [40.6 (5.9) %]. After 12 weeks, body fat percentage increased by 0.3 (1.1) % in the IG and 0.26 (1.1) % in the CG which resulted in a non-significant between-group difference (*ƞ^2^_p_* = 0.0, *p* = 0.94). IG and CG did not differ significantly in their 6MWT performance at baseline (*p* = 0.29). After 12 weeks, both groups improved their covered distance [IG: 23.8 (42.1) m, CG: 17.2 (30.6) m] leading to a non-significant difference in change between groups (*ƞ^2^_p_* = 0.01, *p* = 0.67). Median perceived health scores of IG [3.5 (1)] and CG [4 (1)] were in the upper end of the scale at baseline and did not differ significantly. Neither IG (*r* = 0.31, *p* = 0.28) nor CG (*r* = 0.15, *p* = 0.56) showed significant changes in their perceived health at t1. Similarly, no significant difference for general life satisfaction scores between IG [8 (1)] and CG [7.5 (2)] was found at t0. Changes within IG (*r* = 14, *p* = 0.62) and CG (*r* = 0.35, *p* = 0.19) from t0 to t1 were not significant. Table 2 displays data of both groups at t0 and t1 as well as changes within and between groups.

**Table 2:** Primary, secondary, and exploratory endpoints of both groups before and after the intervention period.

	t0 (baseline)	t1 (after 12 weeks)	Change within groups	Difference of change between groups	*p*	ƞ^2^_p_
**Primary endpoint**						
Muscle (kg) CG (*N* = 14) IG (*N* = 12)	22.2 (3.9)	22 (3.9)	−0.16 (0.51)	0.57 [0.15–1]	**0.01**	0.24
	20.6 (3.6)	21 (3.4)	0.42 (0.54)			
**Secondary endpoints**						
TE (kg) CG (*N* = 13) IG (*N* = 12)	46.2 (19.1)	51.3 (15.6)	5.1 (9.6)	5.8 [−2.2 to 13.7]	0.15	0.09
	32.7 (14.9)	43.6 (15)	10.9 (9.6)			
TF (kg) CG (*N* = 13) G (*N* = 12)	41.5 (16.6)	43.1 (14.5)	1.6 (6.3)	3.1 [−3.2 to 9.4]	0.32	0.04
	29.6 (13.3)	34.3 (13.3)	4.7 (8.8)			
UPush (kg) CG (*N* = 13) IG (*N* = 12)	54.9 (16.6)	61.4 (16.2)	6.6 (12.6)	3.7 [−6.8 to 14.1]	0.48	0.02
	57.9 (12.4)	68.1 (26.9)	10.2 (12.6)			
UPull (kg) CG (*N* = 12) IG (*N* = 12)	46.1 (12.6)	52.4 (9.9)	6.3 (8.7)	6.9 [−2 to 15.8]	0.12	0.11
	52.1 (14.6)	51.5 (16.8)	−0.6 (12.1)			
**Exploratory endpoints**						
YBTl score CG (*N* = 13) IG (*N* = 12)	84.7 (6.1)	88.1 (6.1)	3.4 (6.2)	0.1 [−6.4 to 6.5]	0.99	< 0.001
	93.9 (12.9)	97.4 (8.7)	3.5 (9.3)			
YBTr score CG (*N* = 13) IG (*N* = 12)	84.9 (7.9)	88.5 (5.4)	3.6 (6.6)	0.5 [−4.2 to 5.1]	0.84	0.002
	92.8 (9.9)	96.8 (7.7)	4.1 (4.3)			
6 MWT (m) CG (*N* = 13) IG (*N* = 10)	619.9 (70.9)	637.1 (59.7)	17.2 (30.6)	6.6 [−24.8 to 38.1]	0.67	0.01
	586.6 (74.9)	610.4 (80.4)	23.8 (42.1)			
Body fat (%) CG (*N* = 14) IG (*N* = 12)	40.6 (5.9)	40.9 (6.1)	0.26 (1.07)	0.04 [−0.9 to 0.9]	0.94	< 0.001
	35.3 (6.6)	35.6 (6.3)	0.3 (1.13)			
Perceived health^1^ CG (*N* = 14) IG (*N* = 12)	4 (1)	4 (0.25)	0 (0)		0.56	0.15
	3.5 (1)	4 (1)	0 (0)		0.28	0.31
General life satisfaction^2^ CG (*N* = 14) IG (*N* = 12)	7.5 (2)	8 (1.25)	0 (1)		0.19	0.35
	8 (1)	7 (1)	0 (1.75)		0.62	0.14

Muscle mass, strength, Y-Balance test scores, 6-min walking test, and body fat percentage values for t0, t1, and change within groups are expressed as mean (standard deviation). Differences in change between groups are presented as mean [95% confidence interval]. ƞ^2^_p_ is given for effect size. Perceived health score and general life satisfaction score values for t0, t1, and change within groups are expressed as median (inter quartile range). Pearson’s *r* was calculated for effect size.

CG, Control group; IG, Intervention group; kg, kilograms; m, meters; SD, Standard deviation; TE, Trunk extension; TF, Trunk flexion; UPush, Upper body push; UPull, Upper body pull; YBTl, Y-Balance test left side; YBTl, Y-Balance test right side; and 6MWT, 6-min walking test.

^1^A score from 1 to 5 can be achieved.

^2^A score from 0 to 10 can be achieved.

The bold values indicate that the difference was statistically significant.

## 4 Discussion

The results of the present 12-week pilot study suggest that RES could be an effective training concept for the WHP to improve muscle mass in women with sedentary occupations. Changes in muscle mass were remarkable, considering that women lose about 1.1 kg of muscle mass per decade, with the decline accelerating after age 45 (13). In the intervention group (IG: Nt_0_ = 15, Nt_1_ = 12), muscle mass improved by 0.42 (0.54) kg (+2%) whereas the control group (CG: Nt_0_ = 15, Nt_1_ = 14) lost 0.16 (0.51) kg (-0.7%), which resulted in a large significant between-group change (*p* = 0.01, *ƞ^2^_p_* = 0.24). Considering exclusively the muscle gain per intervention time, these results are not uncommon. In a systematic review, Hagstrom et al. estimated muscle mass gains of 1.45 kg (+3.3%) in healthy women after approximately 15 weeks of RT. However, none of the included studies followed an approach comparable to RES, but rather a more time-consuming conventional strength training routine with mean loads of 70% of the one-repetition maximum (1 RM) (8, 39). Nevertheless, moderate improvements in muscle mass can also be achieved with lower loads (≤ 60% of 1 RM) (40). The current study supports this notion and is in line with results of a RES intervention conducted by Perkin et al. (22). They investigated the effectiveness of a 28-day RES intervention (10 min of lower body bodyweight exercises every day) in older adults (65–80 years) and found improvements in leg lean mass (+1%) and thigh muscle cross-sectional area (+2%). Similar to the recent study, Perkin et al. (22) instructed participants to perform as many repetitions as possible (AMRAP) for each resistance exercise. This training approach allowed to accumulate a high weekly volume even though the volume per training session remained low. Furthermore, it is to assume that muscle protein synthesis was sufficiently increased due to the high intensity of effort elicited by the AMRAP modality. According to Burd et al. (41), even training with very light loads (30% of 1 RM) can stimulate muscle protein synthesis to a comparable extent as training with 90% of 1 RM, as long as the intensity of the effort is high. With the RES routine, participants could have benefited from the muscle protein synthesis response frequently throughout the intervention (42, 43). All in all, this could have partially compensated for the disadvantages of the lower training loads.

With regard to strength, even higher loads (> 80–85% of 1 RM) are recommended to maximally recruit muscle fibers and induce optimal strength gains (44). In the current study, no significant between-group changes were found although the IG showed greater changes in strength than the CG for TE [IG: +10.9 (9.6) kg vs. CG: + 5.1 (9.6) kg], TF [IG: + 4.7 (8.8) kg vs. CG: + 1.6 (6.3) kg], and UPush [IG: + 10.2 (12.6) kg vs. CG: + 6.6 (12.6) kg]. A contrasting development occurred in the upper body pull [IG: −0.6 (12.1) kg vs. CG: + 6.3 (8.7) kg]. The contradictory development of upper body pulling strength could be explained by the exercise selection and loading in the present study. Most resistance exercises targeted the leg (e.g., squats), trunk (e.g., good mornings), and upper body pushing (e.g., push-ups) musculature whereas upper body pulling (e.g., reverse flys) was rarely done. Due to the low strength level combined with an elevated BMI (26.5 kg/m^2^) in the IG, bodyweight exercises seemed to provide sufficient stimuli to increase strength in the TE, TF, and UPush movements. On the other hand, with minimal to no equipment (e.g., resistance bands, pull-up bars, and gymnastics rings) opportunities to generate sufficient resistance in pulling movements were limited in the workplace setting. Nonetheless, changes in strength in the current study were comparable to those other studies reported in women after 15 weeks of RT (39). However, given the development of strength in the CG, it must be assumed that positive changes in strength within both groups could be partly attributed to motor learning effects in the BC. Results from Dalichau et al. confirm this assumption. In their study, they observed strength improvements of 2.2–11.4% in a CG that did not receive any intervention between pre- and posttests (45).

Regarding exploratory endpoints, no significant between-group changes were found. Nevertheless, the YBT data provides valuable information for future investigations and practical application of RES. A previous study in middle-aged and older women recommended to consider the YBT as an assessment tool when developing rehabilitation and exercise programs. However, the authors did not provide YBT performance thresholds (e.g., composite score) that could indicate an increased risk of injury during exercise and concluded that further research is needed to determine the clinical utility of this test (46). Regarding other forms of physical activity, there is already evidence of a relationship between YBT performance and the risk of injury. Plisky et al. found that a composite score of <94% is associated with 6.5 times higher injury rates in female basketball players (47). Indeed, this relationship is not directly transferable to sedentary women performing RES but supports the assumption that YBT performance may be an indicator of increased injury risk. In the present study, both groups were below this threshold before the intervention. During the RES, no adverse events occurred indicating that the demands of the training program were appropriate for the participants’ balance. After the intervention period, both groups improved their YBT scores, with the IG ranging above the threshold of 94% for the left and right side that was reported by Plisky et al. (47). Although both groups were provided six practice trials for each movement direction, learning effects might have still occurred and partly explain increased scores in both groups (47). However, albeit the RES did not target improvements in balance, a trend toward greater increases in the IG compared to the CG was noted. As previous studies reported positive correlations between muscular strength and YBT scores, increased strength in trunk, hip, and leg musculature could have contributed the YBT score of the IG (46, 48). Nevertheless, it is to hypothesize that the training stimulus was not adequate to induce significant improvements in balance control after this short-term RES intervention. The same hypothesis possibly applies to the 6MWT since cardiovascular stress was not a desired stimulus of the present training program. Furthermore, while the 6MWT is a valid tool to evaluate the progression in patients with chronic diseases it appeared to be too insensitive to detect improvements after RES in the included participants. Previous research estimated an average learning effect of 27 m between pre- and posttest which likely superimposed any training effects in the present study given the short intervention time and small sample size (49, 50).

There is a relationship between physical health, life satisfaction, and participation in everyday activities (51). In the present study, both groups reported high median general life satisfaction and perceived their health as good. However, insufficient RT could lead to a progressive decline in functional capacity as the participant’s age. This in turn, could impair their ability to perform everyday activities in the long-term and affect their health status (1, 5, 6). Although the WHP does not deal with people in old age but with people of working age, it could still be deduced that the WHP should therefore emphasize the need for RT to maintain functional capacity.

In terms of motivation, Fyfe et al. who conducted an RES intervention in older adults (age = 69.8 ± 3.8 years, 63% women) reported that participants found RES to be an enjoyable and easy to perform training approach that they were likely to continue after the intervention (23). This is consistent with the present observations. After study completion, all participants intended to continue the program, so that RES was permanently integrated in the WHP of the university. These observations are highly relevant with regard to low participation rates among women in RT as they indicate the presence of behavioral maintenance motives (52). Due to the program structure of RES it could be assumed that time efficiency, low difficulty, and direct implementation of RES at the workplace minimized common barriers to exercise participation (7, 14, 15). As our findings remain exploratory, we recommend that future research considers analyzing the potential of RES in terms of behavioral change and maintenance.

Since this study was a pilot-study, some limitations must be considered when interpreting the results. Firstly, study participants were free to choose between IG and CG leading to significant differences in several measured variables at baseline. Furthermore, based on the between-group changes, a greater sample size would be required to reach statistical significance in most endpoints. In terms of body composition, it must be noted that besides an increase in muscle mass, both groups also showed a non-significant increase in body fat percentage. In this regard, it needs to be considered that body composition was assessed via bioelectrical impedance analysis and results should therefore be taken with caution (53, 54). However, physical activity was only assessed in terms of RT participation and nutritional status was not evaluated at all. Therefore, it cannot be ruled out that participants changed their physical activity and eating behaviors throughout the study which in turn could have affected body mass and composition. We therefore recommend that future studies apply a randomized controlled intervention design with a greater sample size and control especially for variables that could affect body composition and performance. Moreover, future studies should include more homogeneous samples in terms of age in order to assess the effectiveness of RES for specific age groups. Lastly, it remains important to determine whether RES are effective and motivating in the long term.

To summarize, although further research is required to confirm the findings of this pilot study, RES could prove to be an effective alternative to conventional RT. According to our observations, RES might be particularly beneficial to improve muscle mass and strength in women who would otherwise be difficult to motivate for RT. As RES require minimal-to-no equipment, are of low difficulty, and require only a few minutes per day, they could be a cost- and time efficient strategy for the WHP.

## Data availability statement

The raw data supporting the conclusions of this article will be made available by the authors, without undue reservation.

## Ethics statement

The studies involving humans were approved by the Institutional Ethics Committee of the University of the Bundeswehr Munich. The studies were conducted in accordance with the local legislation and institutional requirements. The participants provided their written informed consent to participate in this study.

## Author contributions

TB: Conceptualization, Data curation, Formal analysis, Methodology, Visualization, Writing – original draft. CS: Investigation, Writing – review & editing. AS: Conceptualization, Methodology, Writing – review & editing.
